# Effects of Inspiratory Muscle Warm-Up on Physical Exercise: A Systematic Review

**DOI:** 10.3390/biology12020333

**Published:** 2023-02-20

**Authors:** Carolina Cirino, Anita B. Marostegan, Charlini S. Hartz, Marlene A. Moreno, Claudio A. Gobatto, Fúlvia B. Manchado-Gobatto

**Affiliations:** 1 Laboratory of Applied Sport Physiology, School of Applied Sciences, University of Campinas, Limeira 13484-350, SP, Brazil; 2 Postgraduate Program in Human Movement Sciences, Methodist University of Piracicaba, Piracicaba 13400-000, SP, Brazil

**Keywords:** respiration, respiratory muscles, sports

## Abstract

**Simple Summary:**

In physical training, it is common to perform preparatory activities to improve the performance of subsequent exercise. One of these activities is inspiratory exercise, which has been increasingly applied as a muscle-warm-up strategy due to the demands on the respiratory muscles in high-intensity effort. Generally, inspiratory exercise is performed by a device that works as an inspiratory exerciser, imposing resistance to the respiratory muscles in the inspiratory phase by restricting airflow. This resistance is based on the assessment of the individual’s global inspiratory-muscle strength (maximum inspiratory pressure). Our review aimed to systematically review the literature in order to examine the effects of inspiratory muscle warm-up on the inspiratory, metabolic, respiratory and performance parameters of a main exercise performed by athletes and healthy and active individuals, taking into account the protocols applied as well as the equipment used as the inspiratory exerciser. According to the results, the inspiratory-muscle warm-up was mostly performed in two series of thirty inspirations at 40% of the maximum inspiratory pressure, using POWERbreathe^®^ (IMT Technologies Ltd., Birmingham, UK). Our review demonstrated that inspiratory-muscle warm-up can be a practical and simple additional resource to improve performance in high-intensity physical exercise, taking into account its individualized prescription.

**Abstract:**

This study aimed to systematically review the literature to examine the effects of inspiratory-muscle warm-up (IMW) on the inspiratory, metabolic, respiratory and performance parameters of a main exercise performed by athletes and healthy and active individuals. **Methods:** This systematic review included randomized studies in English based on the criteria of the PICOS model. The exclusion criteria adopted were studies that applied inspiratory exercise to: i. promote long-term adaptations through inspiratory training (chronic responses); ii. obtain acute responses to inspiratory load (overload) during and in breaks from physical effort and in an inspiratory-exercise session (acute training effect); iii. evaluate the effects of IMW on participants with cardiorespiratory and/or metabolic disease. **Data Sources:** PubMed, Embase, MedLine, Scopus, SPORTDiscus and Google Scholar (until 17 January 2023). **Results:** Thirty-one studies were selected. The performance and respiratory parameters were the most investigated (77% and 74%, respectively). Positive effects of IMW were reported by 88% of the studies that investigated inspiratory parameters and 45% of those that evaluated performance parameters. **Conclusions:** The analyzed protocols mainly had positive effects on the inspiratory and performance parameters of the physical exercises. These positive effects of IMW are possibly associated with the contractile and biochemical properties of inspiratory muscles.

## 1. Introduction

The respiratory muscles are responsible for the flow of air into (inspiration) and out of (expiration) the lungs, while respiratory demand is associated with the metabolic rate to ensure homeostasis. In intense exercise, the cardiorespiratory and neuromuscular systems undergo several modulations to sustain the effort. Alveolar ventilation must increase proportionally to the consumption of oxygen (O_2_) and the production of carbon dioxide (CO_2_), consequently improving cardiac output. Furthermore, the higher respiratory rate intensifies the work of the respiratory muscles, requiring adjustments to the breathing pattern and increasing muscle recruitment so as to reduce the metabolic cost of breathing. As a consequence, these adjustments increase the demand for blood flow and O_2_ transport to these muscles during effort [1,2]. Due to these physiological challenges, the inspiratory muscles in particular become more intolerant of exercise when the energy demand exceeds the supply [2] since, in fatigue, these muscles consume 10–15% of the total O_2_ [3] and the blood flow corresponds to up to 16% of the cardiac output [4]. Of the muscles activated during inspiration, the main groups are the diaphragm and the external and parasternal intercostals, while the accessory muscles include the sternocleidomastoid, the scalene, the pectoralis major and minor, the serratus anterior and the iliocostalis cervicis [5,6,7], which perform functions related to inspiratory and motor tasks concomitantly. They are more prone to fatigue during main tasks of greater intensity.

In addition to being a type of training used in clinical practice, inspiratory exercise has become an interesting strategy in the sports field, given the importance of the participation of the respiratory muscles in high-intensity efforts [8]. Clinical trials have shown that this resource can reduce blood pressure, since breathing is a modulator of the autonomic nervous system and the baroreflex [9], as well as, helping in the prevention and rehabilitation of diseases that cause respiratory damage, such as dyspnea and exercise intolerance [10,11,12]. In athletes and active and healthy individuals, inspiratory exercise has brought benefits when used chronically in association with long-term training [8,13,14,15,16], or applied acutely to obtain exercise responses with inspiratory overload [17], in a single inspiratory training session [18] or to assist in the recovery from high-intensity efforts [19]. Moreover, inspiratory exercise has been widely used as a preparatory activity for physical exercise. This is known as the inspiratory-muscle warm-up (IMW) [20,21,22,23,24], which is the particular focus of this systematic review. The application of specific previous activities can provide positive effects for physical exercises by controlling the intensity and duration of previous stimuli, as well as recovery between tasks [25]. The exercise performed in the warm-up before a more intense effort can be a resource for post-activation potentiation, whose effects can improve physical performance [26,27].

The first investigation of IMW was based on the hypothesis that a specific warm-up would affect the global strength of this musculature [28]. After confirming this hypothesis, several studies sought to assess the effects of this intervention not only on the overall strength of the inspiratory muscles [29,30,31,32,33], but also on sports performance, especially in high-intensity efforts, taking into account metabolic and respiratory parameters [20,21,22,23,24,29,31,34,35,36,37,38].

Although the literature has reported the positive effects of IMW on athletes and healthy and active individuals while performing several tasks, there are still gaps regarding the protocols to be applied in relation to the prescription of inspiratory load, as well as the characteristics of the warm-up protocol, such as the number of repetitions (breaths), pauses between efforts and the equipment used as the inspiratory exerciser. Since IMW has been used as a strategy to improve performance, it seems pertinent to discuss the subject in a systematic review, providing robust information both for its proper prescription and for the indication of new paths for future investigations. Therefore, this study aimed to systematically review the literature in order to examine the effects of inspiratory-muscle warm-up on the inspiratory, metabolic, respiratory and performance parameters of a physical exercise performed by athletes and healthy and active individuals, taking into consideration the protocols applied as well as the equipment used as the inspiratory exerciser.

## 2. Methods

This systematic review was conducted according to the methodological guidelines of the Preferred Reporting Items for Systematic Reviews and Meta-Analysis (PRISMA) [39]. This systematic review was not recorded.

### 2.1. Eligibility Criteria

This review addressed randomized studies that applied inspiratory exercise as a muscle -warm-up strategy with the aim of improving the performance of subsequent activity. Only English -language studies were considered for the analysis. The exclusion criteria adopted were associated with the types of studies published in the form of an editorials, letters to the editor, comments, reviews, abstracts, lectures or opinion articles. Regarding the types of intervention, we excluded studies that applied inspiratory exercise to: i. promotes long-term adaptations through inspiratory training (chronic responses); ii. obtain acute responses to inspiratory load (overload) during and in breaks from physical effort and in an inspiratory exercise session (acute training effect); and iii. investigate its effects on participants with cardiorespiratory and/or metabolic disease. The definition of the inclusion criteria was based on the PICOS structured-questions model (P—Population; I—Intervention; C—Comparison; O—Outcome; S—Study design; Table 1). Only studies conducted with athletes and healthy and active individuals aged over 18 years without any motor limitations were considered. No restriction was adopted as to the sex of the participants.

### 2.2. Data Sources and Search Strategy

The comprehensive literature search was performed on the following electronic databases PubMed, Embase, MedLine, Scopus and SPORTDiscus and Google Scholar (additional search). Database queries were restricted to the period between 1999 and 17 January 2023. On all electronic databases, the following combinations of terms were applied: “inspiratory” OR “respiratory” AND “warm-up” OR “pre-activation” AND “exercise” OR “performance.”

### 2.3. Quality Assessment

The PEDro (Physiotherapy Evidence Database) scale was used to assess the methodological quality of the studies included in this review [40]. This scale considers 11 items: 1. eligibility criteria specified; 2. random allocation; 3. concealed allocation; 4. groups similar at baseline; 5. subject blinding; 6. therapist blinding; 7. assessor blinding; 8. dropout rate lower than 15%; 9. intention-to-treat analysis; 10. between-group statistical comparisons; 11. point measures and variability data. The classifications of the methodological quality of the studies were determined as poor (scores ≤3), fair (scores 4–5), good (scores 6–8) and excellent (scores 9–10). The quality assessment was not applied as an inclusion criterion.

### 2.4. Risk of Bias Assessment

The risk of bias was assessed according to the Cochrane Collaboration guidelines (Review Manager software, version 5.4.1, Copenhagen, DK. The bias risk was analyzed in the selection, performance, attrition and reporting domains. The judgement was classified as high, low, or unclear risk of bias [41]. The risk-of-bias assessment was not applied as an inclusion criterion.

### 2.5. Data Extraction and Management

The literature search, screening and selection of articles were carried out by two authors independently (CC and ABM). After consulting the databases, the screening was conducted considering the title, abstract and keywords according to the eligibility criteria. Duplicate references were removed. At a different point in the study, the same authors analyzed the texts in full. In cases of divergence, decisions were taken by a third author (FBMG). The two authors used a customized data-extraction form, taking into account the criteria established based on the PICOS model. This form extracted information about the participants (characteristics, sex, age and sample size), IMW protocols (inspiratory load, number of sets and repetitions, interval between sets and between intervention and physical exercise), performance parameters analyzed and results obtained (IMW effects). Regarding the effects of the interventions, the parameters investigated were: inspiratory (variables related to inspiratory measurements), sports performance (variables provided by the tests to determine the sports performance), respiratory (physiological and psychophysiological variables related to ventilatory measurements) and metabolic (variables associated with the metabolic products of the exercise).

## 3. Results

### 3.1. Study Selection

**Figure 1:**
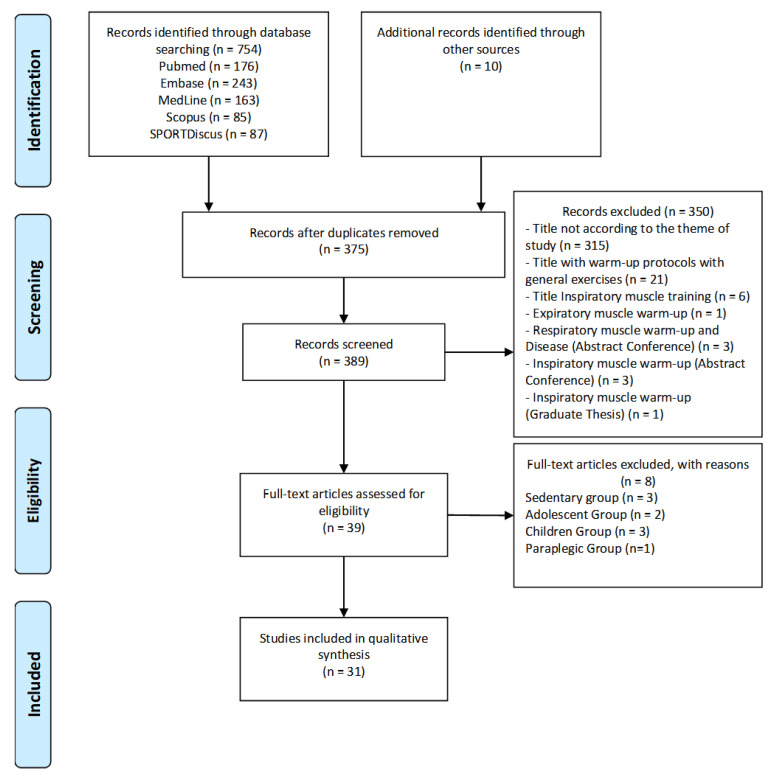
Flow diagram of the study’s screening process.

Initially, a total of 764 studies were selected, of which 176 were from PubMed, 243 were from Embase, 163 were from MedLine, 85 were from Scopus, 87 were from SPORTDiscus and 10 were from Google Scholar (additional database). Next, 375 duplicates were excluded, resulting in 389 studies. Of these, we excluded three-hundred and fifty titles for not being in accordance with the study topic, twenty-one titles with warm-up protocols applied to general exercises, six titles with inspiratory-muscle training, one title with expiratory-muscle warm-up, three titles with respiratory-muscle warm-up associated with disease (conference abstract), three titles with IMW (conference abstract) and one study published as a graduation thesis. From the remaining thirty-nine studies, we extracted three that were applied to sedentary groups, two that were applied to groups of adolescents (aged below 18 years), three that were applied to a group of children and one that was applied to a group of paraplegics. Finally, a total of 31 studies were included in this systematic review (Figure 1).

**Figure 2:**
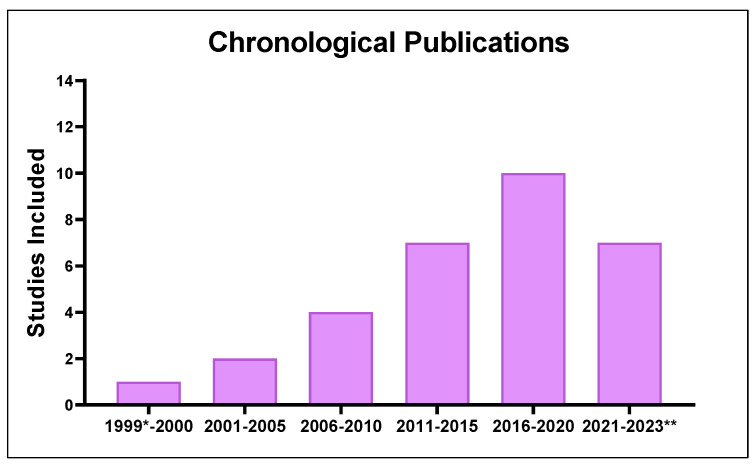
Distribution of published studies on inspiratory muscle warm-up applied to athletes and healthy and active individuals (period analyzed: 1999 to 2023). * First publication. ** Reviewed until 17 January 2023.

Among the selected studies, the chronological analysis of the publications (Figure 2) showed an exponential increase in investigations related to IMW from 1999 (the first study on the subject) to 2020. It should be noted that between 2021 and 2022, 7 studies were published prior to this review (17 January 2023).

### 3.2. Quality Assessment

Table 2 presents the assessment of the methodological quality of the studies. According to the PEDro scale, 77% of the studies had good methodological quality, while the others were classified as being of excellent quality. However, most of the studies did not score on items related to randomization and blinding.

### 3.3. Risk-of-Bias Assessment

**Figure 3:**
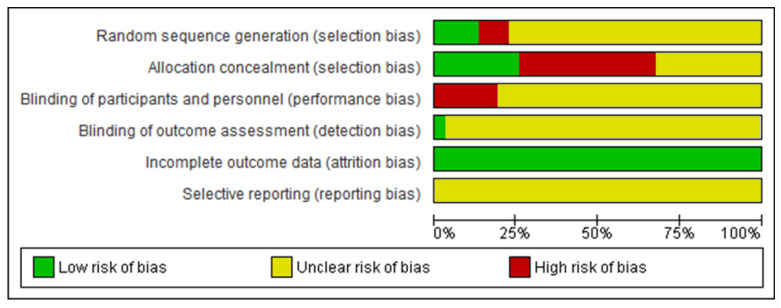
Percentage risk of bias of the studies included in the review.

Figure 3 demonstrates the risk-of-bias analysis of the included studies on a percentage scale. In general, most of the studies omitted methodological information that indicated an unclear risk of bias for all domains, except for incomplete outcome data. This domain has a low risk of bias for all studies because it is used to investigate acute interventions, facilitating data collection. Some of the investigations showed a high risk of bias in domains related to randomization and blinding. Thus, the risk-of-bias analysis raised concerns, since the studies did not explicitly present methodological information.

### 3.4. Study Characteristics

**Figure 4:**
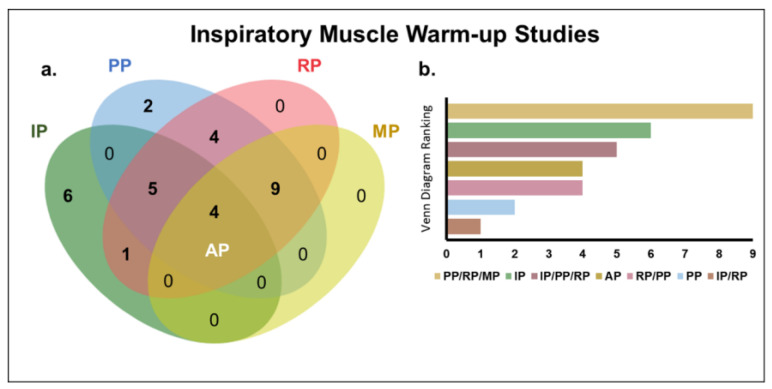
Inspiratory-muscle-warm-up studies. (**a**) Venn diagram [57] of the intersection of studies on the effects of inspiratory-muscle warm-up in relation to the investigated parameters. (**b**) Ranking of the intersections of the Venn diagram (number of studies evaluating the investigated parameters). All parameters (AP); inspiratory parameters (IP); performance parameters (PP); respiratory parameters (RP); and metabolic parameters (MP).

Table 3 presents the characteristics and effects of the IMW protocols applied prior to a physical exercise. The IMW protocols were characterized by the measure used to determine inspiratory load, the prescription of the volume and intensity of inspiratory exercises, the pause between efforts, the type of intervention and the equipment used. The effects of IMW on inspiratory, performance, respiratory and metabolic parameters were also evaluated. A Venn diagram (Figure 4a) was used to quantify the studies in relation to the parameters investigated. According to Figure 4b, of the thirty-one studies considered in this review, nine simultaneously investigated the effects of the intervention on the performance, respiratory and metabolic parameters, six analyzed only the inspiratory parameters, five addressed the inspiratory, performance and respiratory parameters in the same investigation, four quantified all the parameters investigated, four evaluated the effects of the intervention only on the performance and respiratory parameters, two considered only the performance parameters and one analyzed the inspiratory and respiratory parameters in the same investigation.

**Figure 5:**
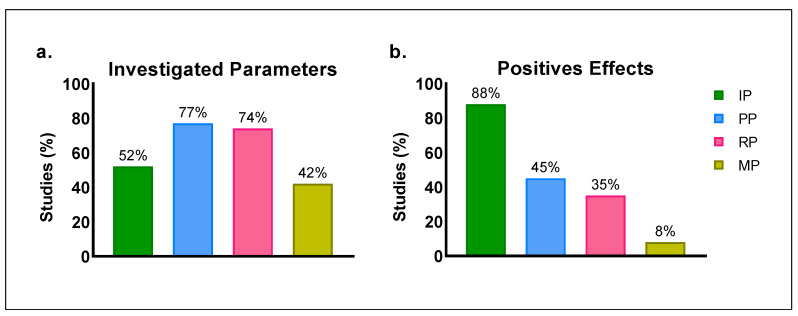
Percentage of studies on the effects of inspiratory-muscle warm-up on inspiratory parameters (IP); performance parameters (PP); respiratory parameters (RP); and metabolic parameters (MP). (**a**) Investigated parameters. (**b**) Positive effects.

Figure 5 shows that the performance and respiratory parameters were the most frequently investigated (77% and 74%, respectively). The positive effects of IMW were reported 88% of the studies that evaluated inspiratory parameters, followed by 45% of those that investigated performance parameters (Figure 5b).

## 4. Discussion

This study investigated the effects of inspiratory exercise applied as a muscle-warm-up strategy prior to physical exercise performed by athletes and healthy and active individuals. According to our analysis, the most frequently investigated factors used to examine the effects of IMW were performance (77%) and respiratory (74%) parameters. The positive effects of IMW were reported in 88% of the studies that evaluated inspiratory parameters and 45% of those that investigated performance parameters. Of the selected studies, the majority concurrently analyzed the effects of IMW on performance, respiratory and metabolic parameters, followed by those that investigated the effects only on inspiratory parameters. Furthermore, most of the investigations that found positive effects of this intervention applied the traditional protocol, consisting of two sets of thirty deep breaths performed using POWERbreathe^®^ (IMT Technologies Ltd., Birmingham, UK), at an inspiratory load of 40% MIP. The assessment of methodological quality and risk of bias was not used as an inclusion criterion. Therefore, all the studies on the subject were analyzed in this review.

Most of the studies covered in this systematic review used maximal inspiratory pressure (MIP) measures to determine effort intensity during IMW, given that these measures are a reference in the assessment of global inspiratory-muscle strength [20,21,22,23,28,29,30,31,32,33,34,35,36,37,38,42,43,44,46,47,48,49,50,51,52,53,54,55], enabling correct individual measurement and guidance on the inspiratory intervention load. The inspiratory-muscle-strength levels were obtained by measuring the maximal inspiratory pressures through the airways or diaphragm [58]. The pressure measurement followed Laplace’s law, which refers to the tension radius of the curvature ratio, which, in this case, was the curvature of the diaphragm dome [59]. In relation to diaphragm tension in particular, the measure used was trans-diaphragmatic pressure (Pdi), which corresponds to the difference between the gastric and esophageal pressures resulting from the passage of catheters via the nasal route to the distal esophagus and stomach [60]. Although this method is the most accurate for assessing the pressure exerted by the diaphragm [61], it is invasive and restricted, as it provides the pressure index of only one inspiratory muscle [60]. Thus, to determine pressure through the airways [56], the MIP is the most commonly used measure in clinical practice [60,62] and in inspiratory-muscle training, since it is a non-invasive method [8,13,14,15,16]. The measure that represents global inspiratory-muscle strength [60,62,63], is obtained using a manovacuometer, by the Mueller maneuver performed against an occluded valve that requires maximum contraction from residual volume to total lung capacity, eliciting maximal isometric effort from the inspiratory muscles [59,62].

Alternatively, more recent studies adopted the use of the S-index (strength-index) measure, which dynamically assesses global inspiratory-muscle strength through the airflow generated in an open system during an inspiratory maneuver [62,64,65]. It should be noted that even though this measure does not represent the MIP, it has proved to be a valid option, capable of providing the necessary measures for the prescription of inspiratory-muscle training [62,64]. In addition, the S-index can be advantageous due to its easy applicability, since the device used to obtain this measure (POWERbreathe^®^, K-Series) can also be employed to perform inspiratory exercises [62]. Although this measure is becoming increasingly popular [62], only the studies by Barnes and Ludge [24] and Silapabanleng et al. [54] adopted it for prescribing inspiratory exercise. While the former [24] applied the protocol before the S-index and 3200-m running tests, the latter [56] used IMW based on S-index prior to a 3-min all-out test on a cycle ergometer.

Regarding the IMW protocol, especially in relation to volume (the number of sets and repetitions) and intensity (the percentage of maximal inspiratory pressure), most studies applied the traditional protocol defined by Volianitis et al. [28], with two sets of thirty breaths at 40% MIP. The use of 40% MIP in the traditional protocol can be justified by the fact that this inspiratory load represents the highest intensity at which inspiratory-muscle fatigue is not induced [44,66]. An IMW protocol with an inspiratory load corresponding to 15% MIP was adopted in some investigations as a placebo, since it did not differ from the protocol without IMW intervention (control) [20,21,22,23,43,48,50,52]. However, other studies that applied two sets of fifteen breaths at 15% MIP showed positive changes, indicating that this inspiratory load can be used to prepare athletes for subsequent high-intensity efforts [36,38].

Of the studies that applied the traditional protocol (2 × 30), most of reported positive effects on MIP measures [28,29,31,32,33,42,44,46,49], in dynamic inspiratory function estimated using different inspiratory loads [20,43], and in improved lung-function parameters [46]. Barnes and Ludge [24] observed an increased S-index in an alternative protocol (1 × 30 breaths at 50% S-index). Regarding the performance parameters, the studies that adopted the traditional protocol found positive effects of IMW on rowing [29,55], badminton [20], intermittent running [31], swimming [22], anaerobic fitness test [23,37,38], maximal running (sprints) [53] and hockey [54]. In these studies, the performance improvement ranged between 2.1% and 34.4% when IMW was applied. However, other investigations that also assessed high-intensity efforts with the intervention of the traditional IMW protocol showed no changes in performance parameters [21,32,34,35,43,47,48,49,52,55]. Although all of these studies applied the same volume and intensity, with intervals between sets of 30 s up to 2 min [23,31,32,34,35,50,52], that is, similar stimuli, the different results found may have been associated with the time between the IMW stimulus and the physical exercise. While Tong and Fu [43] started the physical exercise immediately after the warm-up integrated with the IMW, Lomax et al. [31] waited for 3 min. The other studies did not provide this information in their experimental designs, which may have affected their understanding of the responses to the stimuli caused by the IMW. Other interventions that can provide the post-activation potentiation described in the literature reported short-duration effects of around a few minutes on muscle actions [67]. In this reasoning, it is safe to say that the duration of the IMW’s effects on high-intensity efforts is still unknown; therefore, the time taken to start the physical exercise can affect its effect on performance. Hawkes et al. [44] observed that the positive effects of the traditional protocol of IMW on MIP lost their effectiveness 15 min after the intervention. Moreover, unlike the effort caused by the MIP assessment, the high-intensity stimuli characteristic of sports practice may result in inspiratory-muscle fatigue [1,2], which can affect the duration of the IMW effects.

Other studies sought to assess IMW protocols with different volumes and intensities of inspiratory exercise. Arend et al. [33] assessed the effects of IMW on MIP by applying four different protocols. According to the authors, a protocol with a more intense inspiratory load (60% MIP) and a lower number of breaths (12 inspirations) led to positive effects on global inspiratory-muscle strength. Marostegan et al. [38] also tested IMW at 60% MIP (two sets with fifteen breaths) and observed an increase in mechanical parameters in 30-s all-out tethered running. Merola et al. [51] also applied a protocol at an inspiratory load of 60% MIP (two sets of fifteen breaths) as a preliminary activity to a specific judo test. However, this study did not show changes in performance or respiratory parameters.

It is worth mentioning that the most recent studies, such as those developed by Cirino et al. [36], who evaluated a judo match, and Manchado-Gobatto et al. [37], who evaluated performance in 30-s all-out tethered running, brought innovations to the analysis of the effects of inspiratory-muscle warm-up, demonstrating that the complex-networks model allows a more integrative analysis and highlighting the involvement of physiological parameters that are important for performance.

Regarding the type of equipment used as an inspiratory exerciser in the IMW included in the studies, Thurston et al. [45] used one that promotes inspiratory overload by restricting airflow through holes with diameters between 3 and 13 mm. Since the individualized intensity was not determined, this study found no changes in performance, metabolic or respiratory parameters. Although this equipment is low-cost and easy to apply, it has been used on a smaller scale due to the difficulty in controlling the inspiratory load, as it does not have a mechanism to regulate load during breathing [8]. The study by Lomax et al. [31] was based both on the MIP measure used to define the inspiratory load and on the use of PowerLung^®^ (Sports, USA) to control the restriction of air flow through valves and provided the possibility of acting in the inspiratory and expiratory phases [8,68]. Nonetheless, McConnell and Romer [68] do not recommend the use of this equipment in the expiration phase, as the addition of an expiratory load may increase intrathoracic pressure, consequently increasing the potential risks during effort. It is important to highlight that Lomax et al. [31] observed positive effects of IMW both on sports-performance parameters and on MIP measures. The other studies also prescribed inspiratory exercise based on individualized MIP using POWERbreathe^®^ (IMT Technologies Ltd., Birmingham, UK), a device that acts only in the inspiratory phase but has a regulation mechanism, involving a valve that is controlled electronically, which makes it able to maintain load intensity during inspiration [69].

The effects of IMW on MIP and sports-performance parameters [20,28,30,31,32,33,36,43,46] may be associated with the neural control of the inspiratory muscles, since this musculature exerts ventilatory and postural demands [70,71]. The IMW can help improve intra and intramuscular coordination [20,28,31], as the mechanical efficiency of these muscles requires coordinated muscle action [72], determined by the contractile and fatigue properties of the recruited motor units [73].

With a specific emphasis on metabolic and respiratory aspects, some investigations did not report positive effects on parameters related to responses after intense efforts [20,21,22,24,29,32,34,36,55], except for tissue-oxygenation rate in active muscles [21], respiratory rate [35] and dyspnea perception [20,24,43]. These results may be related to inspiratory-muscle fatigue, as previously discussed [74], since high-intensity efforts increase metabolites close to phrenic nerve endings, sensitizing type III and type IV afferent fibers that activate the metaboreflex [75]. This stimulates adrenergic vasoconstriction, redistributing blood flow from active to respiratory muscles [76]. Cheng et al. [21] suggested that the application of the traditional IMW protocol in submaximal exercise performed on a cycle ergometer may have delayed the metaboreflex, mitigating the drop in oxygen saturation. However, this positive effect was not associated with an improved cycling performance [21]. Furthermore, regarding submaximal exercise, Arend et al. [35] found that the effect of IMW at 40% MIP in rowers increased the respiratory rate during effort. The authors did not consider it a negative sign, but rather an opportunity to increase ventilation without causing dyspnea [35]. With respect to the decrease in dyspnea [20,24,43], which can be considered a limiting factor in maintaining effort, it was verified that the positive effects of IMW on high-intensity efforts helped improve performance [43]. It can therefore be concluded from the results that IMW facilitates chest stabilization, improving the mechanical efficiency of ventilation and reducing the sensation of dyspnea [77,78,79].

In our review, the assessment of the methodological quality and risk of bias of the studies indicated strengths and limitations that must be taken into account. The methodological quality of the studies was classified as good and excellent, according to the PEDro scale. This strengthens the description of the characteristics of the protocols and the possible applications of inspiratory exercises for enhancing the subsequent activity. However, most of the studies did not score items related to the randomization-and-blinding process (PEDro Scale). The main concerns raised by the risk-of-bias analysis reflect this judgment. It is noteworthy that this is the first exploratory review on IMW to select all the studies on the subject in order to indicate a direction for future investigations with more methodological details about inspiratory-muscle-warm-up effects.

## 5. Conclusions

Considering the above, the traditional IMW protocol (two sets of thirty inspirations at 40% MIP) was the most frequently applied to athletes and healthy and active individuals. According to the IMW protocols analyzed in this review, there was an improvement mainly in the inspiratory parameters related to MIP (4.0–21.2%) and in the performance of specific tasks (2.1–34.4%). Although the application of other protocols with control over the number of repetitions, inspiratory load and type of intervention also showed favorable effects on the improvement of physical exercise, further investigation is still required. In conclusion, the positive effects of IMW are associated with the efficiency of inspiratory muscles, possibly due to the contractile and biochemical properties of motor units and muscle fibers. We consider that the use of IMW can be a practical and simple additional resource to improve performance in high-intensity physical exercise, taking into account its individualized prescription.

## Figures and Tables

**Table 1:** Inclusion criteria based on the PICOS method.

Population	Intervention	Comparison	Outcome	Study Design
Athletes and healthy and active adults	Inspiratory-muscle warm-up	Experimental condition X Control and/or placebo	Parameters associated with the physical exercise *	Randomized studies

* Performance (field and laboratory tests), inspiratory, metabolic and respiratory parameters.

**Table 2:** Assessment of the methodological quality of studies (PEDro Scale).

Authors (Year)	Items											Total	Quality
	1	2	3	4	5	6	7	8	9	10	11		
Volianitis et al. (1999) [28]	1	0	0	1	0	0	0	1	1	1	1	6	G
Volianitis et al. (2001) [42]	1	1	0	1	0	0	0	1	1	1	1	7	G
Volianitis et al. (2001) [29]	1	0	0	1	0	0	0	1	1	1	1	6	G
Tong and Fu (2006) [43]	1	1	1	1	1	0	0	1	1	1	1	9	E
Hawkes et al. (2007) [44]	1	0	0	1	0	0	0	1	1	1	1	6	G
Lin et al. (2007) [20]	1	1	1	1	1	0	0	1	1	1	1	9	E
Lomax and McConnell (2009) [30]	1	1	0	1	0	0	0	1	1	1	1	7	G
Lomax et al. (2011) [31]	1	1	0	1	0	0	0	1	1	1	1	7	G
Cheng et al. (2013) [21]	1	1	1	1	1	0	0	1	1	1	1	9	E
Johnson et al. (2014) [34]	1	1	0	1	0	0	0	1	1	1	1	7	G
Wilson et al. (2014) [22]	1	1	1	1	1	0	0	1	1	1	1	9	E
Arend et al. (2015) [35]	1	1	0	1	0	0	0	1	1	1	1	7	G
Ohya et al. (2015) [32]	1	1	1	1	1	0	0	1	1	1	1	9	E
Thurston et al. (2015) [45]	1	1	0	1	0	0	0	1	1	1	1	7	G
Arend et al. (2016) [33]	1	1	0	1	0	0	0	1	1	1	1	7	G
Ozdal (2016) [46]	1	1	0	1	0	0	0	1	1	1	1	7	G
Ozdal et al. (2016) [23]	1	1	0	1	0	0	0	1	1	1	1	7	G
Faghy and Brown (2017) [47]	1	1	0	1	0	0	0	1	1	1	1	7	G
Hartz et al. (2017) [48]	1	1	0	1	0	0	0	1	1	1	1	7	G
Ozdal and Bostanci (2018) [49]	1	1	0	1	0	0	0	1	1	1	1	7	G
Richard and Billaut (2018) [50]	1	1	1	1	1	0	0	1	1	1	1	9	E
Merola et al. (2019) [51]	1	1	0	1	0	0	0	1	1	1	1	7	G
Richard and Billaut (2019) [52]	1	1	1	1	1	0	0	1	1	1	1	9	E
Tong et al. (2019) [53]	1	1	0	1	0	0	0	1	1	1	1	7	G
Avci et al. (2021) [54]	1	1	0	1	0	0	0	1	1	1	1	7	G
Arend et al. (2021) [55]	1	1	0	1	0	0	0	1	1	1	1	7	G
Barnes and Ludge (2021) [24]	1	1	0	1	0	0	0	1	1	1	1	7	G
Cirino et al. (2021) [36]	1	1	1	1	1	0	0	1	1	1	1	9	E
Silapabanleng et al. (2021) [56]	1	1	0	1	0	0	0	1	1	1	1	7	G
Manchado-Gobatto et al. (2022) [37]	1	1	0	1	0	0	0	1	1	1	1	7	G
Marostegan et al. (2022) [38]	1	1	0	1	0	0	0	1	1	1	1	7	G

PEDro Scale Items: 1. eligibility criteria specified; 2. random allocation; 3. concealed allocation; 4. groups similar at baseline; 5. subject blinding; 6. therapist blinding; 7. assessor blinding; 8. dropout rate lower than 15%; 9. intention-to-treat analysis; 10. between-group statistical comparisons; 11. point measures and variability data. Quality score = total score (YES = 1). Poor (P): scores ≤3; fair (F): scores 4–5; good (G): scores 6–8; and excellent (E): scores 9–10.

**Table 3:** Description of the IMW protocols. ^a^ POWERbreathe^®^. ^b^ PowerLung^®^. ^c^ Flow-restriction device. ^d^ Not informed.

Authors (Year)	Subjects		IMW Protocol	Physical Exercise	Results
	Population	Age (Years)			
^a^ Volianitis et al. (1999) [28]	23 subjects 12 non-rowers 11 rowers	20 ± 1 20 ± 2	Control: without IMW IMW: 2 × 30 (40% MIP)	MIP assessment	↑ MIP
^a^ Volianitis et al. (2001) [42]	14 healthy subjects	26 ± 3	Control: without IMW IMW: 2 × 30 (40% MIP) (2 min before physical exercise)	Repeated measurement of MIP(after each series)	↓ “Learning effect” between repeated measurements of MIP
^a^ Volianitis et al. (2001) [29]	14 competitive club rowers 7 Male 7 Female	19.9 ± 0.7 20.1 ± 0.9	Control: without IMW IMW: 2 × 30 (40% MIP)	MIP assessment 6-min all-out rowing	<MIP decrease (%) in the post-test ↑ Power (W) ↑ Distance (m) ↓ Dyspnea
^a^ Tong and Fu (2006) [43]	10 healthy male subjects	21.3 ± 1.2	Control: without IMW Placebo: 2 × 30 (15% MIP) IMW: 2 × 30 (40% MIP) IMW performed between specific warm-up activities Physical exercise performed immediately after specific warm-up activities and IMW	Dynamic inspiratory-muscle-function Ttest Yo-Yo intermittent-recovery test	↑ MIP at zero flow ↑ Maximal inspiratory-muscle power ↑ Optimal pressure ↑ Maximal inspiratory flow↑ Maximal rate of pressure development ↓ Dyspnea No changes in performance parameters
^a^ Hawkes et al. (2007) [44]	12 healthy subjects (6 male)	25 ± 9	Control: without IMW IMW: 2 × 30 (40% MIP)	MIP assessment	↑ MIP ↑ EMG activity of the diaphragm and intercostal muscles on the MIP assessment
^a^ Lin et al. (2007) [20]	10 male badminton players	23 ± 2	Control: without IMW Placebo: 2 × 30 (15% MIP) IMW: 2 × 30 (40% MIP)	Dynamic inspiratory-muscle-function test Maximum incremental badminton-footwork test	↑ MIP at zero flow ↑ Maximal rate of pressure development ↑ Distance (m) ↓ Dyspnea ↓ [Lac] (ISO trial)
^a^ Lomax and McConnell (2009) [30]	8 healthy and active subjects (7 females and 1 male)	29.1± 6.3	Control: without IMW IMW: 2 × 30 (40% MIP) (60 s pause)	MIP assessment	↑ MIP
^b^ Lomax et al. (2011) [31]	12 healthy and semi-professional male football players	24.6 ± 1.3	Control: without IMW IMW: 2 × 30 (40% MIP) (60 s pause) (3 min before physical exercise)	MIP assessment Yo-Yo intermittent-recovery test	↑ MIP ↑ Distance (m) No changes in respiratory parameters
^a^ Cheng et al. (2013) [21]	10 female soccer players	19.9 ± 1.4	Control: without IMW Placebo: 2 × 30 (15% MIP) IMW: 2 × 30 (40% MIP)	2 sets of 6-min cycling exercises 6 × 10 s sprints	No changes in performance, metabolic or respiratory parameters, except ↓ ∆ TSI
^a^ Johnson et al. (2014) [34]	10 trained competitive road cyclists	32 ± 9	1: Without any preliminary activity 2: Control without IMW 3: IMW-2 × 30 (40% MIP) (30s pause)	10-kilometer-cycling time-trial tests	No changes in performance, metabolic or respiratory parameters
^a^ Wilson et al. (2014) [22]	15 elite swimmers (9 male)	21.2 ± 1.6	1: Control without IMW 2: Only IMW-2 × 30 (40% MIP) 3: IMW-2 × 30 (15% MIP) 4: IMW-2 × 30 (40% MIP)	100-m freestyle-sprint time trial	↓ Time No changes in metabolic or respiratory parameters
^a^ Arend et al. (2015) [35]	10 competitive male rowers	23.1 ± 3.8	Control: Without IMW IMW: 2 × 30 (40% MIP) (2 min pause)	Submaximal-intensity rowing test (90% VO_2_ max)	No changes in performance, metabolic or respiratory parameters, except ↓ breathing frequency
^a^ Ohya et al. (2015) [32]	10 healthy and active male subjects	25.1 ± 4.8	Placebo: 2 × 30 (15% MIP) IMW: 2 × 30 (40% MIP) (60 s pause)	5 × 5-s cycling sprints (25-s active recovery)	↑ MIP post-IMW No changes in performance, metabolic or respiratory parameters
^c^ Thurston et al. (2015) [45]	11 recreationally active male subjects	24.9 ± 4.2	Airflow restriction Control: Without IMW Low: 2 × 30 (13-millimeter opening) Medium: 2 × 30 (8-millimeter opening) High: 2 × 30 (3-millimeter opening) (60 s pause)	Cycling test to exhaustion (85% VO_2_max) Pulmonary-function testing (spirometry)	No changes in performance, metabolic or respiratory parameters
^a^ Arend et al. (2016) [33]	10 healthy male subjects	26.4 ± 4.1	Control: Without IMW 1: IMW—2 × 30 (15% MIP) 2: IMW–2 × 30 (40% MIP) 3: IMW—2 × 12 (60% MIP) 4: IMW—2 × 6 (80% MIP) (5 min before physical exercise)	MIP assessment	↑ MIP (IMW at 40 and 60% MIP)
^a^ Ozdal (2016) [46]	26 healthy male subjects	26.31 ± 4.39	Control: Without IMW Placebo: 2 × 30 (15% MIP) IMW: 2 × 30 (40% MIP)	MIP assessment Pulmonary-function testing (spirometry)	↑ MIP ↑ Pulmonary function
^a^ Ozdal et al. (2016) [23]	30 field hockey players	20.5 ± 2.0	Control: Without IMW IMW: 2 × 30 (40% MIP) (2 min pause)	Wingate test	↑ Performance parameters
^a^ Faghy and Brown (2017) [47]	9 healthy and active male subjects	26.4 ± 9.1	1: Control without IMW 2: Only IMW 2 × 30 (40% MIP) 3: Only placebo (5-min breathing using a sham device) 4: IMW 2 × 30 (40% MIP) 5: Placebo (5-min breathing using a sham device)	2.4-kilometer-running timetrials (25-kilogram thoracic load) MIP Assessment	↓ MIP post physical exercise (no difference among protocols)No changes in performance, metabolic or respiratory parameters
^a^ Hartz et al. (2017) [48]	14 female handball athletes	19 ± 1	Control: Without IMW Placebo: 2 × 30 (15% MIP) IMW: 2 × 30 (40% MIP)	Yo-Yo endurance test	No changes in performance, metabolic or respiratory parameters
^a^ Ozdal & Bostanci (2018) [49]	30 male elite field hockey players	20.50 ± 1.98	Baseline: MIP/MEP values Control: Without IMW IMW: 2 × 30 (40% MIP) (2-min pause)	MIP/MEP assessment Incremental test with cycle ergometer (VO_2_ peak)	↑ MIP/MEP (comparison with baseline) ↑ Respiratory parameters
^a^ Richard and Billaut (2018) [50]	7 elite male long-track speed skaters	23.4 ± 3.3	Protocols combined with chronic ischemic preconditioning Placebo: 2 × 30 (15% MIP) IMW: 2 × 30 (40% MIP) (60-s pause)	600-m ice-skating time-trials	No changes in performance or respiratory parameters
^a^ Merola et al. (2019) [51]	11 judo athletes	22.3 ± 2.3	Control: Without IMW Placebo: 2 × 15 (15% MIP) IMW: 2 × 15 (60% MIP) (with one breath immediately after each movement during specific warm-up activities)	Special judo fitness test MIP and MEP assessment	No changes in inspiratory, performance or respiratory parameters
^a^ Richard and Billaut (2019) [52]	8 elite speed skaters (5 Male)	21.4 ± 3.5	Placebo: 2 × 30 (15% MIP) IMW: 2 × 30 (40% MIP) (60 s pause)	3000-m ice-skating time-trials	No changes in performance or respiratory parameters
^a^ Tong et al. (2019) [53]	9 male college athletes (soccer and handball)	20.6 ± 0.9	Control: Without any preliminary activity IMW: 40% MIP (IMW was performed simultaneously with four exercises for the trunk musculature) Interventions were performed between exercises on the treadmill	2 × intermittent efforts on a non-motorized treadmill at progressive speeds up to all-out speed (15-min pause) MIP assessment Sport-specific-endurance plank test (trunk-muscles strength)	Accelerated recovery of MIP and trunk-muscle strength ↑ Performance parameters (sprints) No changes in metabolic or respiratory parameters in the recovery period
^d^ Avci et al. (2021) [54]	30 male hockey players	21.50 ± 2.98	Control: Without IMW Placebo: 2 × 30 (5% MIP) IMW: 2 × 30 (40% MIP) (60-s pause)	Hockey Tests: Hockey drag-flick and shot-performance test Goal and scoring Drag-flick-performance test Hit-performance test	↑ Drag-flick and shooting performance
^a^ Arend et al. (2021) [55]	10 high-level male rowers	23.1 ± 3.8	Control: Without IMW IMW: 2 × 30 (40% MIP) (2-min pause)	Submaximal-intensity rowing test (90% VO_2_max)	No changes in performance or respiratory parameters (VO_2_ kinetics)
^a^ Barnes and Ludge (2021) [24]	17 middle-distance runners: 10 Male 7 Female	20.3 ± 1.5 20.2 ± 1.3	Control: 1 × 30 (30 slow protracted breaths against 3 cm of H20 resistance) IMW: 1 × 30 (50% S-index)	Inspiratory-muscle-function test (S-index) 3200-m-run-performance trial	↑ Performance parameters ↑ S-index No changes in respiratory parameters, except ↓ Dyspnea
^a^ Cirino et al. (2021) [36]	10 male judo athletes	22 ± 1	Control: Without IMW Placebo: 2 × 15 (15% MIP) IMW: 2 × 15 (40% MIP) (60-s pause) (2 min before physical exercise)	Judo match—4 min Recovery post-combat	↑ Technical-tactical parameters No changes in metabolic or respiratory parameters in the recovery period
^a^ Silapabanleng et al. (2021) [56]	26 healthy males subjects	19–23	Control: Without IMW IMW: 2 × 30 (40% S-index) Physical exercise performed immediately after IMW	3-min all-out test in cycle ergometer	↓ Heart rate at the third minute of the test No changes in performance parameters or rate of perceived exertion (RPE)
^a^ Manchado-Gobatto et al. (2022) [37]	15 physically active young men	23 ± 1	Control: Without IMW IMW: 2 × 15 (40% MIP) (60-s pause) (2 min before the physical exercise)	30-s all-out tethered running on a non-motorized treadmill Recovery post-test	↑ Absolute (W) and relative power (W.kg^−1^) No changes in metabolic or respiratory parameters at rest or in the recovery period
^a^ Marostegan et al. (2022) [38]	16 physically active young men	23 ± 1	Control: Without IMW 1: IMW-2 × 15 (15% MIP) 2: IMW-2 × 15 (40% MIP) 3: IMW-2 × 15 (60% MIP) (60-s pause)(2 min before physical exercise)	30-s all-out tethered running on a non-motorized treadmill Recovery post-test	↑ Relative power (W.kg^−1^) and force (N.kg^−1^) in IMW at 15, 40 and 60% MIP ↑ Velocity (m.s^−1^) in IMW at 60% MIP) No changes in metabolic or respiratory parameters in the recovery period
